# Deutscher Expertenkonsens zur PD-L1-Testung („programmed cell death ligand 1“) in der perioperativen Systemtherapie des muskelinvasiven Blasenkarzinoms

**DOI:** 10.1007/s00120-024-02416-1

**Published:** 2024-08-26

**Authors:** G. Niegisch, C. Bolenz, C. Doehn, G. Gakis, A. Hartmann, H. Müller-Huesmann, H. Reis, F. Roghmann, K. Schwamborn, K. Tiemann, M. Retz

**Affiliations:** 1 Klinik für Urologie, Universitätsklinikum und Medizinische Fakultät der Heinrich-Heine-Universität, Düsseldorf, Deutschland; 2 Centrum für Integrierte Onkologie (CIO) Düsseldorf, CIO Aachen-Bonn-Köln-Düsseldorf, Düsseldorf, Deutschland; 3 Klinik für Urologie und Kinderurologie, Universitätsklinikum Ulm, Ulm, Deutschland; 4 Urologikum Lübeck, Lübeck, Deutschland; 5 Klinik für Urologie, Universitätsklinik/Poliklinik und Medizinische Fakultät der Martin-Luther-Universität Halle-Wittenberg, Halle, Deutschland; 6 Urologische Praxis Celle, Celle, Deutschland; 7 MVZ im MediCo für Hämatologie und Onkologie, Innere Medizin, Palliativmedizin, Paderborn, Deutschland; 8 Dr. Senckenbergisches Institut für Pathologie, Universitätsklinikum Frankfurt, Frankfurt, Deutschland; 9 Translationale Uro-Onkologie am Prostatakarzinomzentrum, Marien Hospital Herne – Universitätsklinikum der Ruhr-Universität Bochum, Herne, Deutschland; 10 Institut für Pathologie, Universitätsklinikum rechts der Isar der Technischen Universität München, München, Deutschland; 11 Institut für Hämatopathologie Hamburg, Hamburg, Deutschland; 12 Urologische Klinik und Poliklinik, des Universitätsklinikum rechts der Isar der Technischen Universität München, München, Deutschland; 13 Medizinische Fakultät, Klinik für Urologie, Heinrich-Heine-Universität, Moorenstr. 5, 40225 Düsseldorf, Deutschland

**Keywords:** PD-L1-Testung, Immun-Checkpoint-Inhibitor, Perioperative Systemtherapie, Muskelinvasives Blasenkarzinom, Urothelkarzinom, PD-L1 testing, Immune checkpoint inhibitor, Perioperative systemic therapy, Muscle invasive bladder carcinoma, Urothelial carcinoma

## Abstract

Das Rezidivrisiko von Patienten mit einem muskelinvasiven Harnblasenkarzinom (MIBC) nach radikaler Zystektomie ist abhängig vom pathologischen Tumorstadium. Ein hohes Risiko weisen insbesondere Patienten mit einer lymphonodalen Metastasierung (pN+), lokal fortgeschrittenem (≥pT3) oder residuellem muskelinvasivem Tumor trotz neoadjuvanter Chemotherapie auf. Aktuell nimmt die Bedeutung einer adjuvanten Therapie mit Immun-Checkpoint-Inhibitoren (ICI) im Rahmen perioperativer systemtherapeutischer Konzepte zu. Die Indikationsstellung bei dem derzeit in der Europäischen Union zugelassenen PD-1-Inhibitor („programmed cell death 1“) Nivolumab erfordert die Expressionsbestimmung des PD-L1 („programmed cell death ligand 1“)-Proteins mittels Immunhistochemie im Tumorgewebe. Mit Fokus auf MIBC-Patienten mit hohem Rezidivrisiko ergeben sich neue Fragen in Bezug auf die Durchführung und Interpretation der PD-L1-Testung. Eine interdisziplinäre Expertengruppe aus Deutschland hat relevante Fragestellungen aus klinisch-pathologischer Sicht diskutiert und praxisnahe Handlungsempfehlungen erarbeitet, die die Implementierung einer validierten und qualitätsgesicherten PD-L1-Testung entlang der zugelassenen Indikationen im klinischen Alltag erleichtern sollen.

## Abstract (en)

The risk of recurrence in patients with muscle invasive bladder cancer (MIBC) after radical cystectomy depends on the pathological tumor stage. In particular, patients with lymph node metastasis (pN+), locally advanced (≥pT3), or residual muscle invasive tumor despite neoadjuvant chemotherapy are at high risk. Currently, the importance of adjuvant therapy with immune checkpoint inhibitors is increasing in the context of perioperative systemic therapeutic concepts. The indication for the PD‑1 inhibitor nivolumab currently approved in the European Union requires testing of PD-L1 (programmed cell death ligand 1) protein expression by immunochemistry in tumor tissue. Focusing on MIBC patients at high risk of recurrence, new questions arise regarding the implementation and interpretation of PD-L1 testing. An interdisciplinary group of experts from Germany has discussed relevant issues from a clinicopathological point of view and developed practical recommendations to facilitate the implementation of validated and quality-assured PD-L1 testing for the approved indications in daily clinical practice.

## Hintergrund

In Deutschland erkrankten im Jahr 2018 mehr als 18.000 Personen neu an einem invasiven Harnblasenkarzinom (ICD-10: C67). Männer erkranken häufiger als Frauen und etwa 6000 Patienten versterben jährlich an der Erkrankung [1]. Ungefähr 25 % der Patienten weisen bereits bei Diagnosestellung ein muskelinvasives Blasenkarzinom (≥ pT2) auf („muscle-invasive bladder cancer“, MIBC; [2]) und bis zu 30 % aller Patienten mit zuvor lokal begrenztem Blasentumor entwickeln innerhalb von 5 Jahren nach radikaler Zystektomie eine systemische Tumorprogression [3, 4].

Bei muskelinvasiven Tumoren ist die radikale Zystektomie mit Anlage einer Harnableitung ein Grundbaustein der Therapie. Präoperativ wird bei etwa der Hälfte der MIBC-Patienten das tatsächliche Tumorstadium klinisch unterschätzt („understaging“), bei weiteren 20 % der Patienten wird es überschätzt („overstaging“; [5]). Bei Patienten mit organbegrenztem Tumor (≤ pT2) führt die alleinige Operation zu 5‑Jahres-Gesamtüberlebensraten (OS) von etwa 72 %. Im Gegensatz dazu haben Patienten mit einem lokal fortgeschrittenen Tumor eine schlechte Prognose. Bei Infiltration des perivesikalen Fettgewebes (pT3) liegt das 5‑Jahres-OS bei 43 %, bei Infiltration des umgebenden Gewebes (pT4) nur noch bei 28 %. Bei Befall der lokalen Lymphknoten (pN+) lebt nur noch jeder fünfte Patient nach 5 Jahren [4]. Um die onkologische Effektivität der radikalen Zystektomie zu verbessern, sollte diese im Rahmen eines multimodalen Behandlungskonzepts durchgeführt werden. Perioperativ werden hier Cisplatin-basierte Chemotherapien im neoadjuvanten oder adjuvanten Ansatz sowie ICI im adjuvanten Setting eingesetzt. Neue perioperative Behandlungskonzepte im Sinne einer perioperativen Radio(chemo)therapie oder Radio(immun)therapie sind Gegenstand aktueller Studien.

## Aktueller Therapiestandard

Da eine perioperative Systemtherapie das OS verbessern kann, empfiehlt die S3-Leitlinie Harnblasenkarzinom jedem geeigneten Patienten mit einem MIBC (≥ pT2) eine neoadjuvante Cisplatin-basierte Polychemotherapie als Standard anzubieten. Bei Patienten nach alleiniger Zystektomie und postoperativem Nachweis eines lokal fortgeschrittenen Blasentumors (≥ pT3) und/oder pelvinen Lymphknotenbefalls (pN+) ist eine adjuvante Cisplatin-basierte Chemotherapie indiziert [6]. Viele Patienten sind allerdings aufgrund von Kontraindikationen nicht für eine perioperative Cisplatin-basierte Chemotherapie geeignet, dazu gehört als häufigste Ursache eine eingeschränkte Nierenfunktion mit einer glomerulären Filtrationsrate von < 60 ml/min [7]. Während metastasierten, Cisplatin-ungeeigneten Patienten im palliativen Setting eine Carboplatin-basierte Chemotherapie angeboten werden kann, gilt diese Therapievariante nicht für die perioperative Systemtherapie im kurativen Ansatz. Prospektive Daten zeigen keinen Vorteil für den perioperativen Einsatz einer Carboplatin-haltigen Systemtherapie, in der palliativen Therapie ist das Tumoransprechen einer Carboplatin-basierten deutlich schlechter als das einer Cisplatin-basierten Therapie [6]. Die Etablierung der Immuncheckpoint-Inhibitoren (ICI) im metastasierten und fortgeschrittenen Stadium des Harnblasenkarzinoms führte daher zur Initiierung zahlreicher Studien, in denen die Immuntherapie im perioperativen Einsatz, insbesondere bei Cisplatin-ungeeigneten Patienten, als Monosubstanz oder in Kombination mit klassischen Zytostatika bzw. Antikörper-Wirkstoff-Konjugaten untersucht wurde [6].

## Neue Optionen im adjuvanten Setting

In der S3-Leitlinienversion 2.0 liegen keine Therapieempfehlungen für 2 Hochrisikoblasentumorgruppen vor [3].

1. Patienten, die nach alleiniger radikaler Zystektomie ein lokal fortgeschrittenes Tumorstadium und/oder eine pelvine lymphogene Metastasierung (pT ≥ 3 und/oder pN+) aufweisen, haben ein hohes Rezidivrisiko. Die S3-Leitlinie empfiehlt in dieser Situation eine adjuvante Cisplatin-basierte Polychemotherapie [3] Allerdings ist eine Subgruppe von Patienten postoperativ nicht für eine adjuvante Cisplatin-basierte Chemotherapie geeignet.
2. Eine weitere Problemgruppe betrifft Patienten, die trotz neoadjuvanter Cisplatin-basierter Chemotherapie nach radikaler Zystektomie weiterhin ein muskelinvasives Tumorstadium und/oder pelvine Lymphknotenmetastasen (pT ≥ 2 und/oder pN+) aufweisen.

Für beide Hochrisikopopulationen konnten in der S3-Leitlinie bislang noch keine Therapieempfehlungen ausgesprochen werden [3].

In der multizentrischen, doppelblinden, randomisierten und placebokontrollierten Phase-III-Studie CheckMate 274 (NCT02632409) wurden 2 Patientengruppen (*n* = 709) eingeschlossen: Bei der ersten Gruppe handelte es sich um Cisplatin-ungeeignete Patienten mit einem lokal fortgeschrittenen Tumor und/oder einer lymphogenen Metastasierung (pT ≥ 3 und/oder pN+), bei denen eine radikale chirurgische R0-Resektion eines muskelinvasiven Urothelkarzinoms der Harnblase oder des oberen Harntraktes erfolgt war. Die zweite Patientengruppe hatte bereits eine neoadjuvante Chemotherapie erhalten mit folgender chirurgischer R0-Resektion und postoperativ weiterhin Nachweis eines muskelinvasiven Urothelkarzinoms und/oder einer lymphogenen Metastasierung (pT ≥ 2 und/oder pN+; [8]). Damit sind beide oben genannte Risikopopulationen in dieser klinischen Studie abgebildet. Geprüft wurde die adjuvante Gabe von Nivolumab 240 mg alle 2 Wochen für bis zu 1 Jahr vs. Gabe einer verblindeten Placeboinfusion. Primäre Endpunkte waren das krankheitsfreie Überleben („disease-free survival“, DFS) in der Gesamtpopulation sowie bei Patienten mit einer positiven PD-L1-Expression mit einem Tumor Proportion Score (TPS) ≥ 1 %.

Das mediane DFS in der Gesamtpopulation betrug im Prüfarm 20,8 Monate (95 %-Konfidenzintervall [KI]: 16,5–27,6) und im Kontrollarm 10,8 Monate (95 %-KI: 8,3–13,9). Die Hazard Ratio (HR) betrug 0,70 (98,22 %-KI 0,55–0,90). Für das mediane DFS konnte mit der adjuvanten Immuntherapie mit Nivolumab ein statistisch signifikanter Vorteil nachgewiesen werden (*p* < 0,001). Bei Patienten mit einem positiven PD-L1-Status betrug die DFS-Rate nach 12 Monaten im Prüfarm 67,2 % (95 %-KI: 58,4–74,5) und im Kontrollarm 45,9 % (95 %-KI: 37,1–54,2). Die HR lag bei 0,55 (98,72 %-KI: 0,35–0,85; *p* < 0,001). In einer weiteren Subgruppenanalyse zeigte sich zudem ein Vorteil für Patienten, die eine neoadjuvante Chemotherapie vor radikal chirurgischer Operation erhalten hatten. Die HR für das DFS betrug bei dieser Subgruppe 0,53 (95 %-KI: 0,39–0,72), während bei Patienten ohne neoadjuvante Vortherapie eine HR von nur 0,91 beschrieben wurde (95 %-KI: 0,69–1,21; [8]). Im erweiterten Follow-up (Median: 36,1 Monate) betrug das mediane DFS mit Nivolumab 22 vs. 10,9 Monate mit Placebo (HR: 0,71; 95 %-KI: 0,58–0,86) sowie 52,6 vs. 8,4 Monate bei positivem PD-L1-Status (HR: 0,52; 95 %-KI: 0,37–0,72; [9]). Hinsichtlich des OS wurden kürzlich erste Daten im Rahmen eine Kongresspräsentation vorgestellt. Hier betrug im erweiterten Follow-up (intention-to-treat (ITT): Median: 36,1 Monate, PD-L1-positive Population: 23,4 Monate) das mediane OS mit Nivolumab 69,5 vs. 50,1 Monate mit Placebo (HR: 0,76; 95 %-KI: 0,61–0,96) in der Gesamtpopulation. Bei Patienten mit positivem PD-L1-Status war das mediane OS in beiden Gruppen noch nicht erreicht, aber auch hier zeigte sich mit einer HR von 0,56 (95 %-KI: 0,36–0,86) ein deutlicher Vorteil für die Patienten, die mit Nivolumab behandelt wurden [10]. Studiendaten zum OS sind noch nicht verfügbar. Zusammenfassend ist die CheckMate-274-Studie die erste Studie mit positiven Ergebnissen für eine Immuntherapie mit Nivolumab im adjuvanten Ansatz für 2 Hochrisikogruppen. Auf Basis dieser Daten ist Nivolumab seit April 2022 zur adjuvanten Behandlung des MIBC zugelassen, sofern die Tumorzellexpression von PD-L1 bei hohem Rezidivrisiko nach radikaler Resektion in der Immunhistochemie ≥ 1 % (TC ≥ 1 % bzw. TPS ≥ 1 %) beträgt [11]. Zur Bewertung der PD-L1-Expression können sowohl der TPS als auch der TC-Score herangezogen werden.

Den Ergebnissen einer repräsentativen Umfrage zur Versorgungssituation beim Harnblasenkarzinom in Deutschland (Therapiebeginn in Q4/2019–Q1/2020) zufolge wurde der PD-L1-Status hierzulande in der klinischen Routine bei 53,4 % der metastasierten Patienten sowie bei 26,9 % der Patienten mit MIBC erhoben [12]. Mit der Zulassung von Nivolumab in der adjuvanten Situation des muskelinvasiven Urothelkarzinoms (MIBC) nach radikaler Zystektomie hat sich das Spektrum der PD-L1-testpflichtigen Indikationen nochmals vergrößert. Nachfolgend werden praxisnahe und konsensbasierte Handlungsempfehlungen vorgestellt, die die Implementierung einer validierten und qualitätsgesicherten Biomarkertestung entlang der zugelassenen Indikationen im klinischen Alltag erleichtern sollen. Der Schwerpunkt gilt dabei dem MIBC.

## Bei wem soll eine PD-L1-Diagnostik erfolgen?

### Konsens

Vor Einleitung einer (neo)adjuvanten Therapie bzw. radikalen Zystektomie müssen Patienten mit einem MIBC umfassend über alle Therapieoptionen beim MIBC beraten werden. Dabei müssen auch die potenziell mit der perioperativen Systemtherapie verbundenen Nebenwirkungen adressiert werden. Ein ausführliches Patientengespräch wirkt auch möglichen Informationsdefiziten entgegen. Im Sinne einer optimierten Patientenversorgung soll bei allen Patienten mit einem MIBC das Behandlungskonzept vor Therapiebeginn in einem multidisziplinären Tumorboard festgelegt werden. Zusätzlich sollen folgende Patienten nach radikaler Zystektomie im Tumorboard vorgestellt werden:

- Patienten, die nach alleiniger radikaler Zystektomie ein lokal fortgeschrittenes Tumorstadium und/oder eine pelvine lymphogene Metastasierung (pT ≥ 3 und/oder pN+) aufweisen und eine adjuvante Systemtherapie benötigen.
- Patienten, die trotz neoadjuvanter Cisplatin-basierter Chemotherapie nach radikaler Zystektomie weiterhin ein muskelinvasives Tumorstadium und/oder eine pelvine Lymphknotenmetastasen (pT ≥ 2 und/oder pN+) aufweisen.

**Abb. 1:**
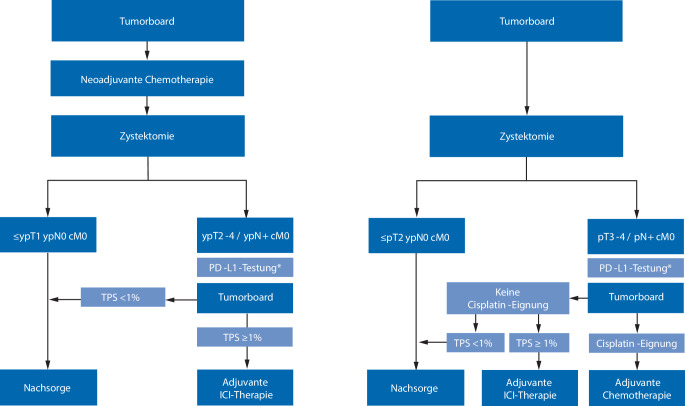
Algorithmus zur perioperativen Systemtherapie in Abhängigkeit von der Vortherapie und dem PD-L1-Expressionsstatus (*Asterisk* spätest möglicher Zeitpunkt; bei negativem PD-L1-Expressionsstatus ggf. Einschluss in klinische Studie prüfen; *PD-L1* „programmed cell death ligand 1“, *ICI* Immun-Checkpoint-Inhibitor [Nivolumab], *TPS* Tumor Proportion Score). (Mod. nach [6])

Eine PD-L1-basierte Biomarkerdiagnostik ist bei Tumoren angezeigt, bei denen sich nach aktuellem Stand der Zulassung Konsequenzen für die Therapieentscheidung ergeben, dazu gehören bei kurativ behandelten Patienten das Tumorstadium (≥ ypT2 bzw. ≥ pT3 und/oder pN+) sowie Cisplatin-ungeeignete Patienten mit einer metastasierten Erkrankung (Abb. 1). Eine Tumorboardvorstellung soll nach Vorliegen des endgültigen histopathologischen Befunds erfolgen.

### Hintergrund

Beim MIBC wird durch die radikale Zystektomie eine Behandlung mit potenziell kurativer Intention angestrebt. Durch die neoadjuvante Kombinationschemotherapie kann beim lokalisierten MIBC das Patientenüberleben gegenüber der alleinigen Zystektomie erhöht werden. Daher sollten Patienten mit MIBC (≥ pT2) bereits im Vorfeld der Zystektomie umfassend über die aktuellen Optionen einer (neo)adjuvanten Chemotherapie beraten werden.

Erfahrungsgemäß erhalten zudem Patienten, die nicht im Tumorboard vorgestellt und evaluiert werden, seltener die Option auf eine perioperative Systemtherapie: Die Ergebnisse einer repräsentativen Umfrage in Deutschland weisen darauf hin, dass eine multidisziplinäre Beratung von Therapieentscheidungen beim MIBC zwar mehrheitlich stattfindet (60,6 %), die Entscheidung aber in etwa 40 % der Fälle allein von den behandelnden Ärzten und ihren Patienten getroffen wird. Etwa die Hälfte der MIBC-Patienten erhielt eine perioperative Systemtherapie, davon 27,5 % neoadjuvant. Wurden die Patienten nicht in einem interdisziplinären Tumorboard vorgestellt, wurde signifikant häufiger auf eine perioperative Systemtherapie verzichtet (Odds Ratio [OR]: 2,43; 95 %-KI: 1,65–3,61; *p* < 0,001; [11]).

Im Tumorboard werden alle beteiligten Disziplinen einbezogen (Urologie, internistische Onkologie, Strahlentherapie, Pathologie, Radiologie/ggf. Nuklearmedizin). Auch die indikationsbezogene PD-L1-Testung sollte in Abstimmung mit einer Tumorkonferenz erfolgen. Je nach den Gegebenheiten vor Ort hat sich auch die reflexartig durchgeführte PD-L1-Testung beim MIBC ≥(y)pT2 bewährt. Ist die PD-L1-Testung nicht direkt in der Pathologie erfolgt, die die primäre Diagnose erstellt, sind insbesondere auch die Operateure (v. a. an rein chirurgischen Kliniken) dazu angehalten, die Empfehlung für die Einholung einer PD-L1-Testung mit in den Operationsbericht bzw. im Entlassungsbrief aufzunehmen.

Der PD-L1-Status bildet eine wichtige zulassungsrelevante Voraussetzung zur Behandlung mit einem ICI. Je nach Indikation ist die Zulassung an den immunhistologischen Nachweis von PD-L1 gekoppelt, welcher hinsichtlich der Scores und Grenzwerte divergiert, die bei der Beurteilung der PD-L1-Expression anzuwenden sind (Tab. 1). Die Zulassung von Nivolumab in der adjuvanten Situation erfolgte beim MIBC mit einer PD-L1-Expression ≥ 1 % auf den Tumorzellen (TC). An dieser Stelle sei angemerkt, dass sich die Zulassung der ICI beim Harnblasenkarzinom i. Allg. auf Urothelkarzinome bezieht – z. T. mit maximal 50 % zulässiger Histologievariante.

**Tab. 1:** Immun-Checkpoint-Inhibitoren (ICI) und PD-L1-Zulassungen („programmed cell death ligand 1“) beim Urothelkarzinom im Überblick. (Mod. nach [13])

	Atezolizumab	Avelumab	Nivolumab	Pembrolizumab
Adjuvant nach chirurgischer R0-Resektion	–	–	PD-L1-TC (bzw. TPS) ≥ 1 %^a^	–
Erstlinie im metastasierten Stadium	PD-L1-IC ≥ 5 %^a^	Unabhängig vom PD-L1-Status	–	PD-L1-CPS ≥ 10^a^
Zweitlinie im metastasierten Stadium (nach Platin)	Unabhängig vom PD-L1-Status	Unabhängig vom PD-L1-Status	Unabhängig vom PD-L1-Status	Unabhängig vom PD-L1-Status

Anmerkungen zum Scoring [3, 13]: Bei der IC (Immune-cells)-Testung wird die PD-L1-Expression auf tumorinfiltrierenden Immunzellen im Tumormaterial bestimmt. Ein IC ≥ 5 % ist ein PD-L1-positiver Status. Bei den TC („tumor cells“) bzw. dem TPS (Tumor Proportion Score) wird das Verhältnis der PD-L1-positiv gefärbten Tumorzellen bezogen auf alle vitalen Tumorzellen angegeben (Angabe in Prozent, bezogen auf das Tumorareal). Ein TC bzw. TPS ≥ 1 % entspricht einem PD-L1-positiven Status. Der CPS (Combined Positive Score) im Tumormaterial ist die Anzahl PD-L1-positiver Tumor- und Immunzellen (nur Lymphozyten und Makrophagen) dividiert durch die Gesamtzahl aller vitalen (d. h. nicht nekrotischen, kernhaltigen) Tumorzellen multipliziert mit 100. Ein CPS ≥ 10 entspricht einem PD-L1-positiven Status

^a^Zulassung vom PD-L1-Status abhängig

## PD-L1-Präanalytik – Was muss der Pathologe wissen und was der Kliniker?

### Konsens

Zum Erhalt optimierter präanalytischer Standards für die PD-L1-Bestimmung ist ein enger interdisziplinärer Austausch zwischen operativer Urologie, konservativer Uroonkologie und Pathologie anzustreben. In biologischer Hinsicht ist insbesondere die potenzielle intratumorale Heterogenität der PD-L1-Expression zu berücksichtigen, die eine sorgfältige Selektion des Untersuchungsmaterials vor dem Hintergrund der aktuellen Therapie- und Tumoranamnese des Patienten erforderlich macht. Im Zweifelsfall empfiehlt sich bei diskrepanten Befunden die Rücksprache mit dem Pathologen. Der Pathologe muss obligatorisch über eventuelle Vortherapien informiert werden, dazu gehören die neoadjuvante Chemotherapie unter Angabe des Therapieschemas, einer BCG (Bacillus Calmette-Guerin)-Therapie oder Bestrahlung sowie ggf. bevorstehende Therapien. Zudem sollten Angaben über Tumor- und Krankheitshistorie sowie die Entnahmestelle der Probe hinreichend mitgeteilt werden (z. B. Primärtumor, Metastase). Zu verwenden ist grundsätzlich das aktuelle Untersuchungsmaterial, um eine möglichst repräsentative Momentaufnahme des immunologischen Tumorstatus zu erhalten – auf archivierte Proben kann und darf zurückgegriffen werden. Die Auswahl des geeigneten Untersuchungsmaterials und Areals obliegt dem Pathologen; für eine externe pathologische Befundung wird in der Regel ein repräsentativer Tumorblock zur Verfügung gestellt.

### Hintergrund

Für die therapeutische Entscheidungsfindung ist der initiale Informationsaustausch mit der Pathologie maßgeblich. Der Pathologe kann grundsätzlich entweder nach Rücksprache mit den Klinikern als Zusatzuntersuchung eine indikationsspezifische PD-L1-Testung (z. B. bei bekannter Nichteignung für eine platinhaltige Chemotherapie) oder eine Reflextestung auf PD-L1 vornehmen, die alle relevanten Scoring-Algorithmen berücksichtigt und als Grundlage für eine Therapieentscheidung dienen kann. Dabei sollten Methoden verwendet werden, mit denen sich die PD-L1-Expression sowohl auf Tumorzellen als auch Immunzellen nachweisen lassen. Die PD-L1-Scoring-Ergebnisse sind sowohl bezogen auf Tumorzellen als auch Immunzellen anzugeben, ebenso sollten die für alle Therapieindikationen relevanten bzw. auf individuelle Anfrage des Klinikers angewandten Auswertungsalgorithmen angegeben werden. Im Befund sollten daher neben den Tumorzellscores (TC, TPS) auch die weiteren Scores für die PD-L1-Expression auf Immunzellen (IC-Score) und dem kombinierten Positivscore (CPS) angegeben werden. Der PD-L1-Klon sollte ebenfalls im Befund mitgeteilt werden (Tab. 2).

**Tab. 2:** Anforderungsprofile zum Informationsaustausch zwischen Klinik/Pathologie

*Angaben des Operateurs bzw. Klinikers an die Pathologie*		
Klinische Diagnose Vortherapien/Therapielinie	⇨	Z. B. Zustand nach neoadjuvanter Therapie/weitere Vortherapien
Datum der Biopsie/Resektion	⇨	Aktuellste Probe verwenden
Materialtyp	⇨	Z. B. Formalin-fixiertes Gewebe
Methode der Probengewinnung	⇨	Z. B. TURB, Resektion Zystektomie
Herkunft/Lokalisation	⇔	Z. B. Metastase vs. Primarius unter Angabe der Lokalisation (z. B. Lebermetastase/LK-Metastase) Ggf. Rücksprache
Anforderung zu PD-L1-Testung	⇔	Wichtig auch bei Versand zur externen Befundung (Operateure in der Pflicht!) Ggf. Rücksprache
Ggf. Information zu geplanter Therapie	⇔	Ggf. Rücksprache
*Befundbericht der Pathologie an die Kliniker*		
Datum der IHC	⇨	–
*PD-L1-Testung*	⇨	Die Auswahl obliegt dem Pathologen (Wahlfreiheit)
Angabe zum Anti-PD-L1-Klon		
Angabe zur IHC-Plattform		
Ggf. Angabe zu LDT		
*PD-L1-Testresultate unter Angabe*	⇔	Ggf. Rücksprache, sonst Reflextestung unter Verwendung aller relevanten Scoringsysteme, ggf. Rücksprache zur Befundinterpretation
der Zellkompartimente (TC, IC, TC und IC)		
Scoringmethode		
Absolutwerte		
Histologische Subtypisierung nach WHO 2022	⇨	Ggf. auf aggressiven Subtyp hinweisen
LVI ja/nein	⇨	Ggf. Information zur prognostischen Relevanz^a^

*IC* Immunzellen, *IHC* Immunhistochemie, *LDT* Laboratory Developed Test, *LK* Lymphknoten, *LVI* lymphovaskuläre Invasion, *TC* Tumorzellen, *TURB* transurethrale Blasenresektion, *PD-L1* „programmed cell death ligand 1“

^a^Vorliegen einer LVI spricht für eine eher ungünstige Prognose nach radikaler Zystektomie: Tumoren, die keine Lymphknotenmetastasen aufweisen, jedoch eine LVI, sind mit einer deutlich schlechteren Überlebensprognose vergesellschaftet als ohne LVI [3, 22]

Weiterhin sind im Befundbericht die verwendeten Färbeantikörperklone und immunhistochemischen Plattformen zu dokumentieren.

Therapien im Vorfeld haben zytologische und histologische Folgen für die Tumorpathologie. Insbesondere kann eine im Vorfeld durchgeführte neoadjuvante Chemotherapie zu einer veränderten PD-L1-Expression führen [14, 15]. Auch kann die PD-L1-Expression je nach Lokalisation des Tumors sowohl intratumoral (z. B. transurethrale Blasenresektion [TURB] vs. radikale Zystektomie) – teilweise auch innerhalb des einen und desselben Schnittpräparats – als auch intertumoral (Primarius vs. Metastase) heterogen ausfallen. Dabei wurde eine höhere intratumorale Heterogenität der PD-L1-Expression in Fernmetastasen berichtet als im Primärtumor [16]. Andere Autoren weisen allerdings auch auf eine Verminderung der PD-L1-Expression und Veränderung des tumorimmunologischen Milieus in Metastasen hin [17]. Die Frage, bei welchem Ausgangsmaterial (Metastase/Lymphknoten, TURB, Zystektomieprobe) die PD-L1-Testung am repräsentativsten ist, konnte bislang nicht abschließend beantwortet werden. In einer immunhistochemischen Vergleichsstudie mit zwei verschiedenen Antikörper-Assays wurde eine größere Variabilität zwischen den gematchten Tumorproben eines Urothelkarzinoms beobachtet als zwischen den Assay-spezifischen Ergebnissen [18].

Kliniker sind darauf hinzuweisen, dass die PD-L1-Expression dynamisch ist und jeweils nur eine immunologische Momentaufnahme darstellen kann. Mögliche Einflussfaktoren umfassen nicht nur intrinsische zelluläre Faktoren, sondern auch den Tumorprogress bzw. das Krankheitsstadium oder das angewandte Therapieschema. Veränderungen der PD-L1-Expression im Verlauf sind nicht nur nach zytotoxischen, zielgerichteten oder immunonkologischen Tumorbehandlungen möglich, sondern auch nach einer operativen Resektionstherapie [19]. In Interventionsstudien, die unter Einsatz von Biomarkertestungen beim Urothelkarzinom erfolgten, wurden aktuelle und frische Tumorproben am therapienaiven Patienten allerdings nur selten (7 %) als Einschlusskriterium vorausgesetzt. Vielmehr waren in ICI-Studien mehrheitlich (67,4 %) archivierte Tumorproben erlaubt [20]. Aufgrund des volatilen Charakters der PD-L1-Expression könnte das Timing der Probenahme eine relevante Rolle bei der Auswertung des PD-L1-Expressionsstatus spielen [20], so dass ggf. auch eine erneute Metastasenbiopsie zur Indikationsstellung für eine immunonkologische Therapie diskutiert werden sollte. Auch in der klinischen Routine ist die Auswahl an Tumorproben meistens begrenzt und es bedarf einer selektiven Auswahl durch den Pathologen. Um eine Erfassung von repräsentativen Tumoranteilen zu gewährleisten, wird vorgeschlagen, dass die Tumorprobe eine Mindestzahl von 100 TC enthalten sollte [17, 21]. Eine besondere Vorbereitung des Probenmaterials ist für die PD-L1-Testung nicht erforderlich; die wesentlichen präanalytischen Prozesse sind mit dem Prozedere bei anderen immunhistochemischen oder molekularen Testverfahren vergleichbar [21].

## PD-L1-Analytik: Welche technischen Aspekte sind zu beachten?

### Konsens

Die Indikation zur gewebebasierten PD-L1-Testung richtet sich nach den aktuellen therapie- und indikationsspezifischen Vorgaben und unterliegt einer hohen Dynamik. Im Unterschied zu den USA besteht in Deutschland Wahlfreiheit bei den verschiedenen Färbeantikörpern und Plattformen in validierten Testsystemen. Grundsätzlich deuten verschiedene Analysen und Konkordanzstudien aber auf eine weitgehende Vergleichbarkeit zwischen den relevanten Testsystemen hin [23, 24].

### Hintergrund

Immunhistochemisch lässt sich die PD-L1-Expression sowohl auf Tumorzellen (TC), als auch auf tumorinfiltrierenden Immunzellen (IC) wie Makrophagen, dendritischen Zellen, Neutrophilen, myeloiden Suppressorzellen und T‑Zellen und/oder B‑Zellen nachweisen, welche sich im intratumoralen und angrenzenden Stroma befinden [25]. In technischer Hinsicht sind die indikationsbezogenen Testpflichten und die damit assoziierten, z. T. sehr unterschiedlichen Auswertungsalgorithmen zu berücksichtigen. So wird zum Einsatz von Pembrolizumab bei Cisplatin-ungeeigneten Patienten mit lokal fortgeschrittenen oder metastasierten Urothelkarzinomen als Nachweis einer positiven PD-L1-Expression ein „Combined Positive Score“ von mindestens 10 gefordert (CPS ≥ 10), für Atezolizumab ein IC-Flächenanteil von mindestens 5 % (IC ≥ 5 %; Tab. 1; [26]).

Fehlen in den Standardantragsformularen der Pathologie wichtige klinische Angaben, bietet die Beschreibung der PD-L1-Expression unter Angabe aller relevanten Auswertungsalgorithmen einen pragmatischen Ansatz (Angabe von TC/TPS, IC-Score und CPS unter Nennung des Antikörperklons und der Färbeplattform; [27]). Innerhalb der indikationsbezogenen Testpflichten und Auswertungsalgorithmen zum PD-L1-Expressionsstatus sind die klinisch relevanten Cut-offs für ein positives Testergebnis sowie die „Interpretationsleitfäden“ und Manuals der Assay-Hersteller zu beachten (PharmDX 28‑8, PharmDx 22C3, SP142).

Weiterhin zeigen die verschiedenen PD-L1-Assays und unterschiedlichen Färbeantikörper ein divergentes Färbeverhalten. Eine deutliche Schwäche für die PD-L1-Detektion auf Tumorzellen wurde für den Antikörperklon SP-142 feststellt, teilweise auch für Immunzellen, wofür der diagnostische Antikörper ursprünglich entwickelt wurde [3, 21]. Unter Berücksichtigung dieser Ausnahme deuten analytische Studien auf generell hohe Übereinstimmungswerte beim Scoring (IC und TC) zwischen den Beobachtern in Bezug auf TC [23, 24]. Eine weitere Validierung der Austauschbarkeit der Assays in größeren Studien ist anzustreben.

Die Validität der PD-L1-Färbung in der Pathologie sollte durch qualitätssichernde Maßnahmen nachgewiesen werden (z. B. Akkreditierung der Pathologie bei der Deutschen Akkreditierungsstelle: DakkS nach DIN ISO 17020, PD-L1-Ringversuche im Rahmen der Qualitätssicherungsinitiative Pathologie QuIP).

## Wie erfolgt die Qualitätssicherung der PD-L1-Testung?

### Konsens

In Anbetracht der zunehmenden Komplexität von Biomarkertest-pflichtigen Therapiealgorithmen beim Urothelkarzinom bedarf es einer kontinuierlichen Verbesserung und Standardisierung der Testverfahren sowie ihrer Auswertung. Die regelmäßige Teilnahme an Ringversuchen ermöglicht pathologischen Laboren und Instituten eine interne und externe Qualitätssicherung durch den Nachweis qualitätsgesicherter Strukturen und Prozesse. In Deutschland ist die PD-L1-Testung in der Fläche etabliert; die Institute nehmen in aller Regel an Ringversuchen teil. Qualitätssichernd wirken sich auch die Vorgaben der Verordnung (EU) 2017/746 über In-vitro-Diagnostika (IVDR) aus, die in jedem Labor – auch in pathologischen Laboren/Instituten – umgesetzt werden müssen. Damit entfällt in den meisten Fällen die Notwendigkeit, im Bereich der PD-L1-Testung Proben extern befunden zu lassen, was sich auch günstig auf das Zeit- und Kostenmanagement auswirken dürfte.

### Hintergrund

Mit der wachsenden Anzahl an diagnostischen und prädiktiven Biomarkern, die immunhistochemische oder molekulare Untersuchungen in der Pathologie erforderlich machen, wird auch die Qualitätssicherung herausfordernder, da die Ergebnisqualität direkten Einfluss auf die Therapieentscheidung hat. Insbesondere wird daher die Teilnahme an externen Qualitätssicherungsprogrammen empfohlen [26]. Hierzulande dienen Ringversuche als zentrales Instrument der internen und externen Qualitätssicherung: Die regelmäßige Teilnahme an Ringversuchen ist fester Bestandteil der Zertifizierungs- und Akkreditierungsverfahren und wird im deutschsprachigen Raum von der „Qualitätssicherungs-Initiative Pathologie“ (QuIP) angeboten, einer gemeinsamen Unternehmung der Deutschen Gesellschaft für Pathologie (DGP) und des Bundesverbands Deutscher Pathologen (BDP) für Ringversuche im Bereich diagnostischer und prädiktiver Immunhistochemie und Molekularpathologie. Die Qualitätssicherungs-Initiative Pathologie bietet kontinuierliche Schulungen sowie Seminare und Trainings zur Vor- und Nachbereitung von Ringversuchen an (https://www.quip.eu/de_DE/trainings). Neben der QuIP bieten zudem weitere Vereinigungen wie z. B. die Europäische Gesellschaft für Pathologie (ESP-EQA) Ringversuche an, die für eine Teilnahme sinnvoll sind. Im Rahmen der Ringversuche werden insbesondere die Färbung und Auswertung beurteilt [28]. Zusätzlich werden alle für Pathologen wesentlichen Kriterien zur PD-L1-Testung und Therapie im PD-L1-Portal der QuIP zusammengefasst (https://www.pdl1portal.eu/; [13]).

## Fazit für die Praxis

- Die PD-L1-Testung („programmed cell death ligand 1“) stellt einen wichtigen Pfeiler in der Therapieplanung des muskelinvasiven Harnblasenkarzinoms (MIBC) dar.
- Die vorgelegten praxisnahen Handlungsempfehlungen sollen die Implementierung einer validierten und qualitätsgesicherten PD-L1-Testung entlang der zugelassenen Indikationen im klinischen Alltag erleichtern.
- Eine neue Indikation in der uroonkologischen Therapielandschaft ist die adjuvante Immuntherapie mit Nivolumab in 2 Patientengruppen mit einem hohen Rezidivrisiko und PD-L1-positivem Status: Cisplatin-ungeeignete Patienten mit einem lokal fortgeschrittenen Tumorstadium und/oder pelviner lymphogener Metastasierung (pT ≥ 3 und/oder pN+) nach erfolgter radikal chirurgischer R0-Resektion eines muskelinvasiven Urothelkarzinoms der Harnblase oder des oberen Harntrakts und Patienten nach neoadjuvanter Chemotherapie und radikal chirurgischer Operation, die weiterhin ein muskelinvasives Tumorstadium und/oder eine pelvine lymphogene Metastasierung zeigen (pT ≥ 2 und/oder pN+).
